# Guizhi Fuling Wan, Chinese Herbal Medicine, Ameliorates Insulin Sensitivity in PCOS Model Rats With Insulin Resistance via Remodeling Intestinal Homeostasis

**DOI:** 10.3389/fendo.2020.00575

**Published:** 2020-08-27

**Authors:** Ying Zhu, Yin Li, Min Liu, XiaoDan Hu, Hongqiu Zhu

**Affiliations:** ^1^School of Clinical Medical, Chengdu University of Traditional Chinese Medicine, Chengdu, China; ^2^College of Medicine and Life Sciences, Chengdu University of Traditional Chinese Medicine, Chengdu, China

**Keywords:** polycystic ovary syndrome, Guizhi Fuling Wan, gastrointestinal microbiome, insulin resistance, homeostasis

## Abstract

Polycystic ovary syndrome (PCOS) is a common endocrine disease with reproductive dysfunction and metabolic disorder in women of childbearing age. Gastrointestinal microbiome contributes to PCOS through mediating insulin resistance. Guizhi Fuling Wan, Chinese herbal medicine, can treat PCOS with insulin resistance (PCOS-IR), but the underlying mechanism is not clear. The aim of this study was to characterize the exact mechanism of Guizhi Fuling Wan action and whether it is related to the regulation of intestinal flora structure. We induced PCOS-IR rat model by means of letrozole sodium carboxymethyl cellulose (CMC-na) solution combined with high-fat emulsion administration and randomly divided it into blank control group (K), model control group (M), low dose of Guizhi Fuling Wan group (D), middle dose of Guizhi Fuling Wan group (Z), high dose of Guizhi Fuling Wan group (G) and positive drug (Metformin) control group (Y). After 36 days of modeling and treatment, serum and stool samples from all rats were collected for a follow-up analysis. The data display that, compared with K group, elevated testosterone and HOMA-IR, turbulent estrous cycles and polycystic ovaries in M group, indicating the PCOS-IR rat model is successfully established. Increased fasting insulin is associated with higher inflammation(plasma TNF-α, IL-6, and HS-CPR concentration were determined) in M group, and the altered intestinal flora (compared with the K group, in M group the relative abundance of *Alloprevotella* was decreased significantly, while the relative abundance of *Lachnospiraceae UCG-008, Lachnospiraceae NK4A136, Lactobacillus, Ruminiclostridium 9*, and *Ruminococcaceae UCG-003* was increased significantly) induced the secretion of inflammatory markers. On the other hand, Guizhi Fuling Wan can alleviate inflammation, improve insulin resistence: Lower inflammation decreased fasting insulin can be seen in G group compared with M group, this effect is related to the regulating effect of Guizhi Fuling Wan on intestinal flora (in G group, the relative abundance of *Alloprevotella, Ruminococcaceae UCG-003*, and *Lachnospiraceae UCG-008* was increased significantly, compared with M group). This research demonstrates Guizhi Fuling Wan improve insulin resistance in polycystic ovary syndrome with the underlying mechanism of regulating intestinal flora to control inflammation. It would be useful to promote the therapeutic effect of Guizhi Fuling Wan on PCOS-IR.

## Introduction

Polycystic ovary syndrome (PCOS) is a common reproductive endocrine disorders in women of childbearing age, and it is also the leading cause of anovulatory infertility (1). Its etiology is complex and multiple factors contribute to the development of polycystic ovary syndrome. The prevalence of PCOS in women of reproductive age ranges from 6 to 20% around the world (2), and the incidence rate is 5.6% in China (3). Clinical and/or biochemical hyperandrogenemia (HA), oligo-anovulation (OA), and polycystic ovarian morphology (PCOM) are three main characteristics of PCOS, the diagnosis of PCOS, according to the Rotterdam Consensus criteria (2003), requires two of the above (4). In addition to reproductive dysfunction, PCOS patients often have metabolic disturbances characterized by insulin resistance (IR) and display an inflammatory state, with an increased risk of developing type 2 diabetes (T2D), dyslipidemia, hypertension, and cardiovascular disease (5–9). Besides, PCOS-related insulin resistance and chronic inflammation is independent of obesity (10, 11). Insulin resistance affecting about 44–70% of PCOS patients (12), is believed to be a key factor in the development of PCOS, closely related to chronic inflammation (1, 13, 14).

The gut is the body's largest organ for digestion and detoxification where hundreds of millions of bacteria inhabit. Abnormal gut microflora is closely related to the occurrence of diseases. Pedro and Bryan performed a cohousing study using a letrozole-induced PCOS mouse model, the results suggest that dysbiosis of the gut microbiome may contribute to PCOS (1). In human studies, scholars found the structure of intestinal flora in PCOS patients is significantly different from that in normal women and a subtle link can be seen between the metabolic abnormality of polycystic ovary syndrome and the gut microbiome changes (1, 15, 16). In 2004 Bäckhed et al. had confirmed that gut microbiota is associated with the onset of insulin resistance (17). The change of gut microbiota can directly and indirectly affect immune cells in the gut to mediate insulin resistance, the indirect effects are achieved via gut microbial products (LPS, metabolites, and SCFAs) which are closely related to inflammation (18). So the above studies suggest that gut microbiota contributes insulin resistance with the underlying mechanism of the inflammation induced by gut microbiota.

In China, Traditional Chinese medicine is still one of the main methods to treat PCOS. Originated from the *Synopsis of the golden chamber* · *treatment of pregnancy diseases* written by Zhang Zhongjing, Guizhi Fuling Wan, a traditional Chinese Medicine formula composed of *Gui Zhi, Fu Ling, Tao Ren, Bai Shao, Dan Pi*, has been widely used to treat multiple gynecological diseases (19). According to the basic theory of traditional Chinese medicine, it can activate blood, resolve blood stasis, and dissipate phlegm. Based on modern medical researches, Guizhi Fuling Wan has an excellent anti-inflammatory effect and the ability to improve insulin resistance, usually used to treat chronic pelvic inflammatory disease and polycystic ovary syndrome (20–23). *Gui Zhi, Fu Ling, Tao Ren, Bai Shao*, the compositions of Guizhi Fuling Wan, are frequently used to treat PCOS, Besides, *Bai Shao* contains paeoniflorin which is an effective compound in the treatment of PCOS (24), and the efficacy of paeoniflorin in improving insulin resistance has been proved in both mammals and vitro experiments (25, 26). Researchers also found paeoniflorin plays an active role in the regulation of beneficial bacteria in intestinal flora. We speculate that Guizhi Fuling Wan containing *Bai Shao* could also regulate intestinal flora and its efficacy in improving PCOS-IR and PCOS related inflammation is achieved by affecting gut microbiota. In this study, we choose letrozole combined with high fat diet to induce a PCOS-IR rat model (27, 28) to investigate the assumptions we mentioned above.

## Materials and Methods

### Animals and Animal Husbandry

Six-week-old specific-pathogen free (SPF) level female Sprague-Dawley (SD) rats [Laboratory animal license number: SCXK (chuan) 2015-030] with-body mass of 180–200 g were provided by Chengdu Dossy Experimental Animals Co., Ltd. All animals raised in SPF laboratory of experimental animal center of Chengdu University of TCM and the whole animal experiment operating process in accordance with the Animal Care Committee of Chengdu University of TCM, China (SYXK2018-0126). In this experiment, 72 rats were randomly divided into six groups of 12 rats each, including blank control group (K), model control group (M), low dose of Guizhi Fuling Wan group (D), middle dose of Guizhi Fuling Wan group (Z), high dose of Guizhi Fuling Wan group (G), and positive drug (Metformin) control group (Y). The blank control group rats received a gavage of normal saline once a day (10 ml/kg). Rats in the other groups were given a gavage of letrozole (Jiangsu Hengrui pharmaceutical Co., Ltd., China) at a concentration of 1 mg/kg dissolved in aqueous solution of carboxymethylcellulose (CMC [10 g/L]) and high-fat emulsion (76.9% lard and 23.1% cholesterol) at concentrations of 15 ml/kg once a day. At the same time, rats in D, Z, G groups were administered with Guizhi Fuling Wan (Chengdu Jiuzhitang Jinding Pharmaceutical Co., Ltd., China) at concentrations of 0.31, 0.62, and 1.24 g/kg by gavage, respectively, once a day, Y group rats was performed metformin (Sino-American Shanghai Squibb pharmaceutical Co., Ltd., China) by gavage once a day at a concentration of 270 mg/kg. The entire modeling and administration process lasted successive 35. From the 6 day on, vaginal smears were collected daily. On 35 day all rats fasted at 8 p.m. On 36 day, all rat fecal samples were collected and stored in a stool box (−800°C) for 16S rDNA amplicon sequencing. Then all rats were sacrificed by cervical dislocation. All blood samples and ovarian tissue samples were collected for following analysis.

### Vaginal Smear and Ovarian Morphological Observation

From the 6 day on, vaginal smears were collected daily and the determination of estrous cycle was evaluated microscopically with hematoxylin-eosin (HE) staining. On the 36 day, ovaries were obtained, weighed, fixed in 10% paraformaldehyde for 48 h, and then embedded in paraffin, sectioned at 5 μm, stained with hematoxylin and eosin and analyzed using an Olympus DP73 microscope.

### Biochemical Assays

All rats' fasting plasma glucose (FPG) was measured by bayer blood glucose meter 1455 (Bayer HealthCare LLC). Serum INS, and testosterone (T) was determined by radioimmunoassay (RIA) using RIA kits (Mibio, Shanghai, China). According to calculation formula: $HOMA-IR=\frac{FINS\times FPG}{22.5}$, we used FPG and FINS to calculate Homeostasis model assessment of insulin resistance index (HOMA-IR) which is used to evaluate insulin sensitivity and to observe the efficacy of Guizhi Fuling Wan in improving insulin resistance. Plasma TNF-α were determined using enzyme-linked immunosorbent assay (ELISA) kit (Elabscience Biotechnology, Wuhan, China), plasma TNF-A, IL-6, and HS-CRP were determined using enzyme-linked immunosorbent assay (ELISA) kits [MultiSciences (LiankeBio), Hangzhou, China].

### 16S rRNA Gene Sequencing and 16S rRNA Gene Sequence Analysis

Genomic DNA from rat feces samples was extracted using a specific DNA extraction kit, and then the DNA was detected by 0.8% agarose gel electrophoresis. With the diluted genomic DNA as the template, The 16S rRNA V4 region of the sample was amplified by PCR using 515F (5′-GTGYCAGCMGCCGCGGTAA-3′) and 806R (5′-GGACTACHVGGGTWTCTAAT-3′) (29, 30), three PCR technique repeats were performed for each sample. The resulting amplicons were recovered, purified with the QIA quick Gel Extraction Kit (QIAGEN) and quantitated with the qubit@2.0 Fluorometer (Thermo Scientific). Illumina TruSeq DNA PCR-Free Sample Prep Kit (FC-121-3001/3003) was used for libraries construction and PE250 sequencing was performed using Illumina Hiseq Rapid SBS Kit V2 (FC-402-4023 500 Cycle). According to the overlap relationship between PE reads, the double-ended sequence is spliced into a sequence using FLASH8, that is, Raw Tag. In order to obtain high-quality sequences, it is necessary to carry out quality control on the sequences, and the sequences obtained after quality control are called Clean Tag. UCHIME9 algorithm was used to remove chimerism and Effective Tag was obtained. The following bioinformatics operation is completed using Usearch (version 7.1) and QIIME. In order to facilitate the study of the species composition and diversity information of samples, the taxon in the clustering generation operation of sequences, namely OUT (Operational Taxonomic Units), is needed. OTU cluster analysis is usually performed at a similar level of 97%. Each OTU represents a set of similar sequences, and the OTU abundance table can be obtained through the OTU cluster analysis, which is the basic file for subsequent analysis. To obtain the corresponding classification information of each OTU, UCLUST taxonomy, and SILVA database (Rlease_123) were used to annotate the classification of the representative sequences of OUT. The richness, evenness, and diversity of the microbial community can be reflected by calculating the alpha-diversity index (Simpson index) of the samples.

### Statistical Method

Statistical analysis was performed using IBM SPSS25.0 software. Data were expressed as Mean ± sem. Normality test was performed by Shapiro-Wilk test and homogeneity of variance test was performed by Levene test. One-way ANOVA with LSD's *post-hoc* test was used to determine the significance of the data which is satisfied the normal distribution and the equal variance among groups. If the data is satisfied non-normal distribution and/or unequal variance, Kruskal-Wallis non-parametric tests were used to determine the significance of the data. Because the data is not binormal, Spearman correlation analysis is used to assess the correlation between the data. *P* < 0.05 means the difference is statistically significant.

## Results

### Letrozole and High-Fat Emulsion Administration Resulted in Typical Endocrine and Ovarian Morphology and Function Changes of PCOS

#### Letrozole and High-Fat Emulsion Administration Resulted in Elevated Serum Testosterone Levels

**Figure 1:**
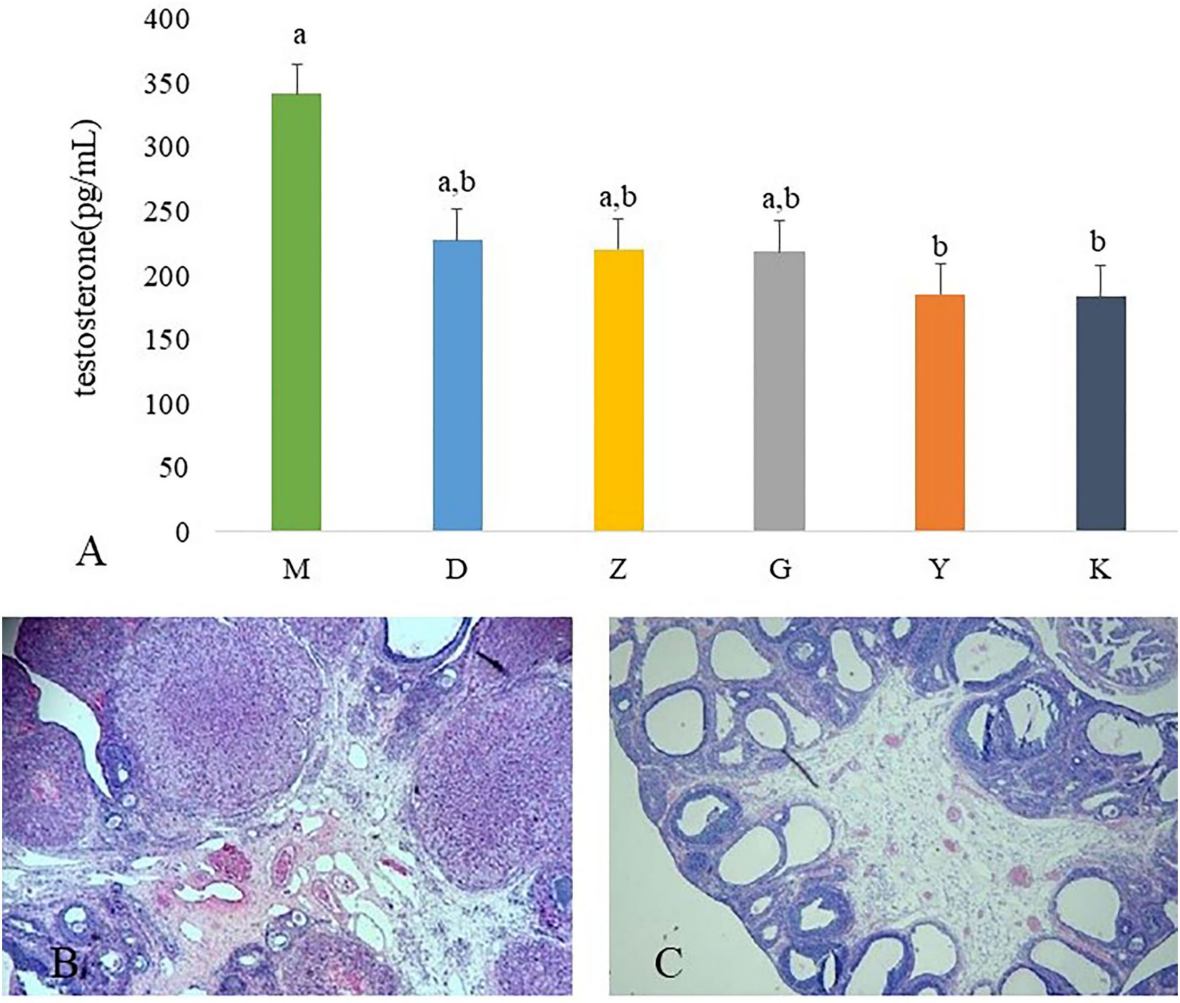
Results of ovarian tissues morphology changes and the plasma concentration of testosterone. The plasma concentration of testosterone in model control group (M), low dose of Guizhi Fuling Wan (L), middle dose of Guizhi Fuling Wan (Z), high dose of Guizhi Fuling Wan (G). Different superscript letters indicate significant differences (*P* < 0.05) in data according to Kruskal-Wallis statistical analysis **(A)**. The ovarian tissue morphology of blank control group (K) **(B)**, the ovarian tissue of model control group (M) **(C)**.

The serum testosterone concentrations was significantly higher in M group rats than those in K group rats. After administration of Guizhi Fuling Wan, the concentration of plasma testosterone is decreased, but without significant difference. Metformin can significantly inhibit testosterone secretion (Figure 1A).

#### Letrozole and High-Fat Emulsion Administration Resulted in Disturbed Polycystic Ovarian Morphology and Estrou Cycle

The ovaries of the K group showed normal morphology (Figure 1B). Rats in M group appeared increased vesicular follicles, atretic follicles, and thin granular cell layer (Figure 1C).

**Figure 2:**
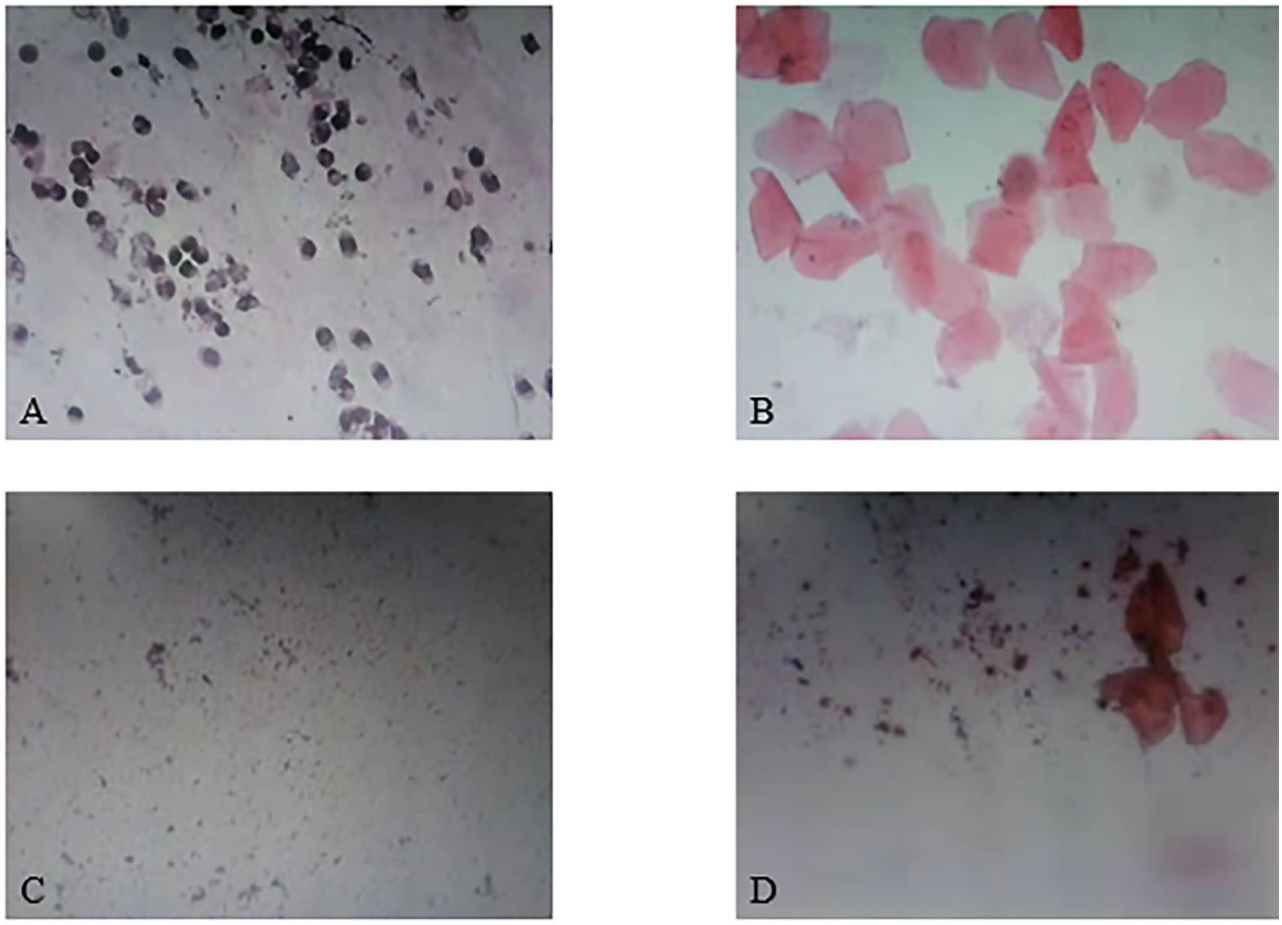
Results of vaginal smear. **(A)** Proestrus. **(B)** Estrus. **(C)** Diestrus. **(D)** Metestrus.

As the results showed, the estrous cycle of rats in the blank control group showed regular periodic changes, while the estrus cycle of rats in the model group was disordered and basically maintained in the diestrus. The vaginal smear in the model group showed a large number of spot-shaped white blood cells and a few patchy keratinocytes (Figure 2C). Guizhi Fuling Wan could improve the estrous cycle of rats, and its effect was similar to that of metformin: the estrus cycle of rats in the low, middle, high dose of Guizhi Fuling Wan groups and the positive drug group basically recovered and showed periodic changes (Figures 2A,B,D).

### Guizhi Fuling Wan Treatment Could Ameliorate IR

Compared with the K group, plasma fasting blood glucose, fasting insulin level, and the insulin resistance index were significantly increased in the M group (*P* < 0.01). Compared with the M group, the plasma fasting blood glucose, fasting insulin level and insulin resistance index of D, Z, G, Y groups rats were significantly decreased (*P* < 0.01; Table 1).

**Table 1:** Fasting blood glucose and insulin levels of rats in each group.

**Group**	**FPG (mmol/L)**	**FINS (mU/L)**	**HOMA-IR**
K	4.34 ± 0.21	25.70 ± 5.60	5.08 ± 1.14
M	6.94 ± 0.41^Δ^	46.95 ± 7.93^Δ^	14.81 ± 2.56^Δ^
D	6.25 ± 0.39^*^	32.71 ± 6.18^*^	9.21 ± 1.80^*^
Z	5.61 ± 0.64^*^	32.24 ± 6.33^*^	8.37 ± 1.61^*^
G	5.31 ± 0.22^*^	31.81 ± 4.09^*^	7.50 ± 0.94^*^
Y	5.51 ± 0.34^*^	31.93 ± 6.80^*^	8.16 ± 1.88^*^

Δ *P < 0.01 data are significantly different vs. blank control group (K)*.

* *P < 0.01 data are significantly different vs. model control group (M)*.

*Blank control group (K), model control group (M), low dose of Guizhi Fuling Wan group (D), middle dose of Guizhi Fuling Wan group (Z), high dose of Guizhi Fuling Wan group (G), and positive drug (Metformin) control group (Y)*.

### Guizhi Fuling Wan Treatment Reduce the Plasma Concentration of Inflammatory Markers to Control Inflammation

**Figure 3:**
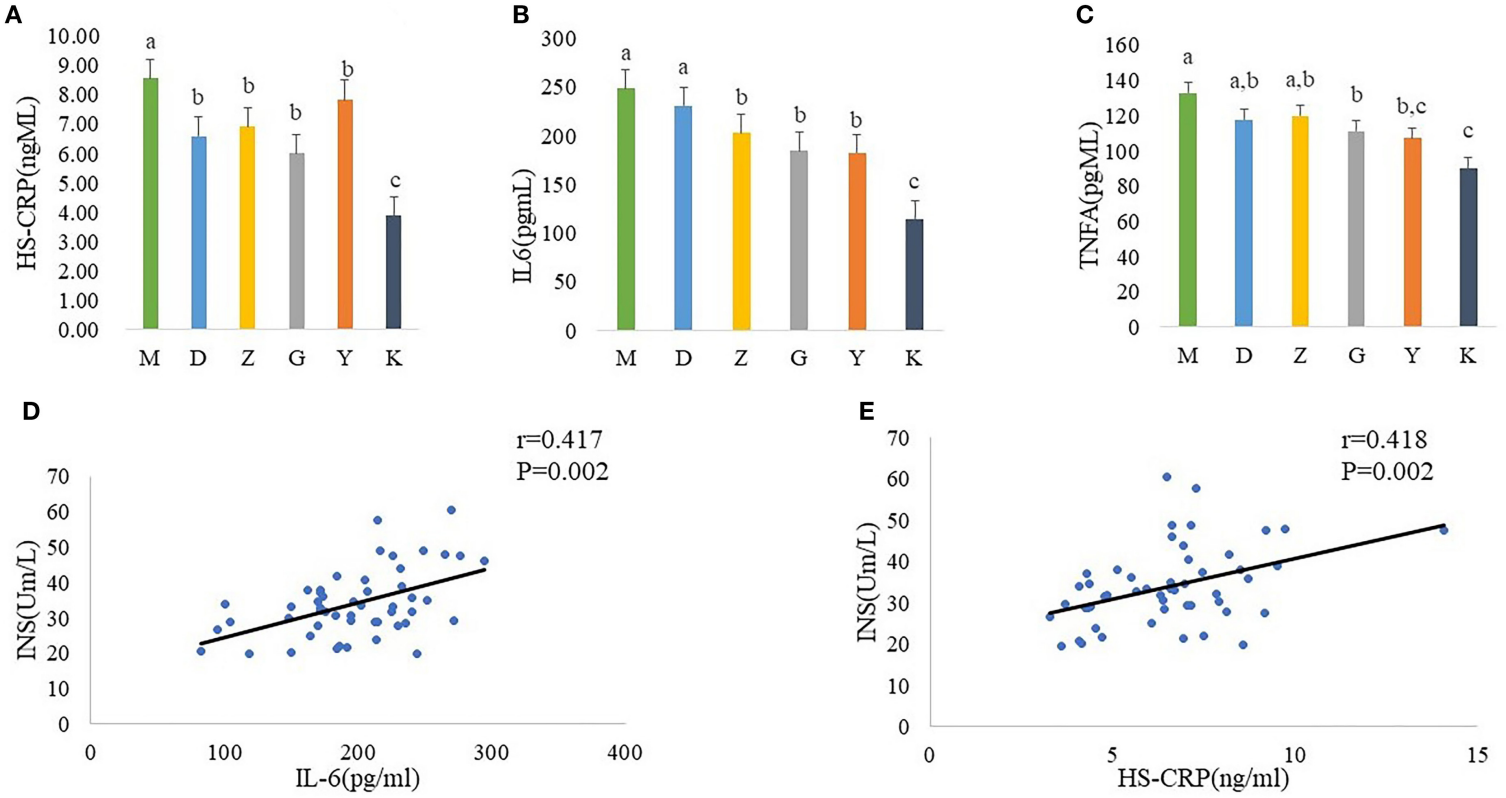
Plasma concentration of HS-CPR **(A)**, IL-6 **(B)**, and TNF-α **(C)** in model control group (M), low dose of Guizhi Fuling Wans (L), middle dose of Guizhi Fuling Wans (Z), high dose of Guizhi Fuling Wans (G). Different superscript letters indicate significant differences (*P* < 0.05) in data according to Kruskal-Wallis and the *post-hoc* ANOVA statistical analysis. Correlation analysis between inflammatory cytokines and fasting insulin: A positive correlation can be seen between inflammatory cytokines (IL-6, HS-CRP) and INS **(D,E)**. The “*r*” refers to the Spearman correlation coefficient, and the *P* < 0.05 indicate differences are significant.

In order to better link the changes in intestinal flora of PCOS model rats with insulin resistance, we measured the serum concentration of HS-CPR, IL-6, and TNF-α. And statistics indicate that a significant increase in plasma concentration of HS-CPR, IL-6, TNF-α was found in M group, compared with K group (Figures 3A–C). Although it did not completely inhibit inflammation, Guizhi Fuling Wan can reduced the release of HS-CPR, IL-6 and TNF-α to alleviate the systemic inflammatory state (Figures 3A–C).

In addition, we further analyzed the correlation between inflammatory cytokines and fasting insulin. The results showed that the serum fasting insulin concentration increased with the increase of il-6 and HS-CRP (Figures 3D,E).

### Alpha Diversity Analysis of Intestinal Flora

Simpson: one of the indices used to estimate microbial alpha diversity, taking into account the abundance and evenness of species. Here the result is 1-D (D is Simpson's Index), and the higher 1-D, the higher α diversity of gut microflora. There was no significant difference in 1-D between the model group and the blank control group (*P* > 0.05). Compared with the model group, the 1-D of D and Z group was significantly lower than that in the model group (*P* < 0.01, *P* < 0.05; Table 2), however, there was no significant difference in 1-D of G and Y group (Table 2).

**Table 2:** Simpson index of intestinal flora of rats in each group.

**Group**	**Case number**	**1-D**
M	11	0.9867 ± 0.0007
D	10	0.9533 ± 0.0058^**^
Z	9	0.9630 ± 0.0023^*^
G	10	0.9862 ± 0.001
Y	9	0.9877 ± 0.0005^ΔΔ^
K	11	0.9647 ± 0.0029

ΔΔ *P < 0.01 data are significantly different vs. K group*.

* *P < 0.05*,

** *P < 0.01 data are significantly different vs. M group*.

*Blank control group (K), model control group (M), low dose of Guizhi Fuling Wan group (D), middle dose of Guizhi Fuling Wan group (Z), high dose of Guizhi Fuling Wan group (G), and positive drug (Metformin) control group (Y)*.

As *Firmicutes, Bacteroidetes*, and *Proteobacteria* are the three main types of bacteria with the highest relative abundance in intestinal flora, the changes in the number of these three bacteria will have a certain impact on the structure and function of intestinal microecology. Compared with M group, there was a decrease can be seen in the number of *Firmicutes, Bacteroidetes*, and *Proteobacteria* in D group (without a significant difference) (Table 3). The number of *Firmicutes* and *Bacteroidetes* in Z group had a slight elevation (Table 3), but the relative abundance of *Proteobacteria* was significantly decrease, compared with M group. So in general, the relative abundance of the three bacteria in D and Z group had decreased, which leading to a decrease in intestinal diversity. In G group, compared with M group, the number of *Firmicutes* was significantly increased, while the *Bacteroidetes* was significantly decreased. A rise and fall can be seen in G group, so the diversity of intestinal flora had no difference between G and M group. We can speculate from the results that the higher the dose of Guizhi Fuling Wan, the higher the α-diversity of intestinal flora.

**Table 3:** The relative abundance of the three main types of bacteria in low, medium, and high dose of Guizhi Fuling Wan group, model group and positive drug group.

	**Classfication**	**M**	**D**	**Z**	**G**	**Y**
Firmicutes	Phylum	0.4578 ± 0.0168	0.4467 ± 0.0117	0.4692 ± 0.0216	0.6082 ± 0.0168^*^	0.5411 ± 0.0097^*^
Bacteroidetes	Phylum	0.4918 ± 0.0195	0.4362 ± 0.0079	0.5071 ± 0.0227	0.3522 ± 0.0204^*^	0.4224 ± 0.0071^*^
Proteobacteria	Phylum	0.0300 ± 0.0033	0.0254 ± 0.0098	0.0119 ± 0.0017^*^	0.0177 ± 0.0019^*^	0.0202 ± 0.0024^*^

Compared with model group

* *P <0.01*.

*blank control group (K), model control group (M), low dose of Guizhi Fuling Wan group (D), middle dose of Guizhi Fuling Wan group (Z), high dose of Guizhi Fuling Wan group (G) and positive drug (Metformin) control group (Y)*.

### Intestinal Flora Abundance Analysis

#### Changes of Intestinal Flora in PCOS-IR Model Rats

**Figure 4:**
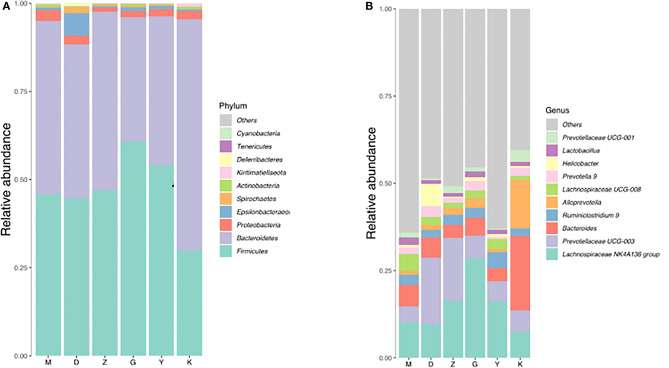
Barplot of relative abundance at phylum level **(A)** and Genus level **(B)**.

Both M group and K group were mainly composed of *Bacteroidetes, Firmicutes*, and *Proteobacteria*. In the M group, *Bacteroidetes* accounted for 49.18%, *Firmicutes* for 45.78% and *Proteobacteria* for 3%. At the phylum classification level, compared with K group, the relative abundance of *Bacteroidetes* was decreased with no significant difference (Figure 4A, Table 4), and the relative abundance of *Firmicutes* and *Proteobacteria* were increased with no significant difference (Figure 4A, Table 4), it is worth mentioning that the *P*-value between the number of *Firmicutes* in M group and K group is just equal to 0.05, which is statistically significant in theory but not in practice. Therefore, we believe that there is no significant difference in the number of *Firmicutes* between group M and group K. At the genus classification level, compared with the K group, the relative abundance of *Alloprevotella* was decreased significantly (*P* < 0.01; Figure 4B, Table 4). While the relative abundance of *Lachnospiraceae UCG-008, Lachnospiraceae NK4A136, Lactobacillus, Ruminiclostridium 9*, and *Ruminococcaceae UCG-003* was increased significantly (*P* < 0.01; Figure 4B, Table 4). The remaining bacteria had no significant difference between the K and M group (Table 4).

**Table 4:** Relative abundance of intestinal flora of rats in model group and blank group.

	**Classification**	**M**	**K**	**Tendency**
Firmicutes	Phylum	0.4578 ± 0.0168^*^	0.2995 ± 0.0283	↑
Bacteroidetes	Phylum	0.4918 ± 0.0195	0.6551 ± 0.0293	↓
Proteobacteria	Phylum	0.0300 ± 0.0033	0.0229 ± 0.0015	↑
Lachnospiraceae NK4A136 group	Genus	0.1013 ± 0.0085^*^	0.0742 ± 0.0111	↑
Prevotellaceae UCG-003	Genus	0.0465 ± 0.0056	0.0614 ± 0.0052	↓
Bacteroides	Genus	0.0610 ± 0.0058	0.2136 ± 0.0461	↓
Ruminiclostridium 9	Genus	0.0287 ± 0.0013^*^	0.0216 ± 0.0036	↑
Alloprevotella	Genus	0.0112 ± 0.0011^*^	0.1383 ± 0.0068	↓
Lachnospiraceae UCG-008	Genus	0.0489 ± 0.0042^*^	0.0124 ± 0.0021	↑
Prevotella 9	Genus	0.0175 ± 0.0025	0.0226 ± 0.0047	↓
Helicobacter	Genus	0.0077 ± 0.0014	0.0053 ± 0.0011	↑
Lactobacillus	Genus	0.0216 ± 0.0020^*^	0.0121 ± 0.0010	↑
Prevotellaceae UCG-001	Genus	0.0143 ± 0.0026	0.0335 ± 0.0030	↓
Ruminococcaceae UCG-003	Genus	0.0187 ± 0.0021^*^	0.0042 ± 0.0010	↑
Bifidobacterium	Genus	0.0012 ± 0.0004	0.0006 ± 0.0002	↑
Enterobacteriaceae	Family	0.0014 ± 0.0005	0.0031 ± 0.0010	↓

Compared with the blank control group

* *P < 0.01*.

*Blank control group (K), model control group (M), low dose of Guizhi Fuling Wan group (D), middle dose of Guizhi Fuling Wan group (Z), high dose of Guizhi Fuling Wan group (G), and positive drug (Metformin) control group (Y)*.

#### Gut Flora Is Related With Inflammation

**Figure 5:**
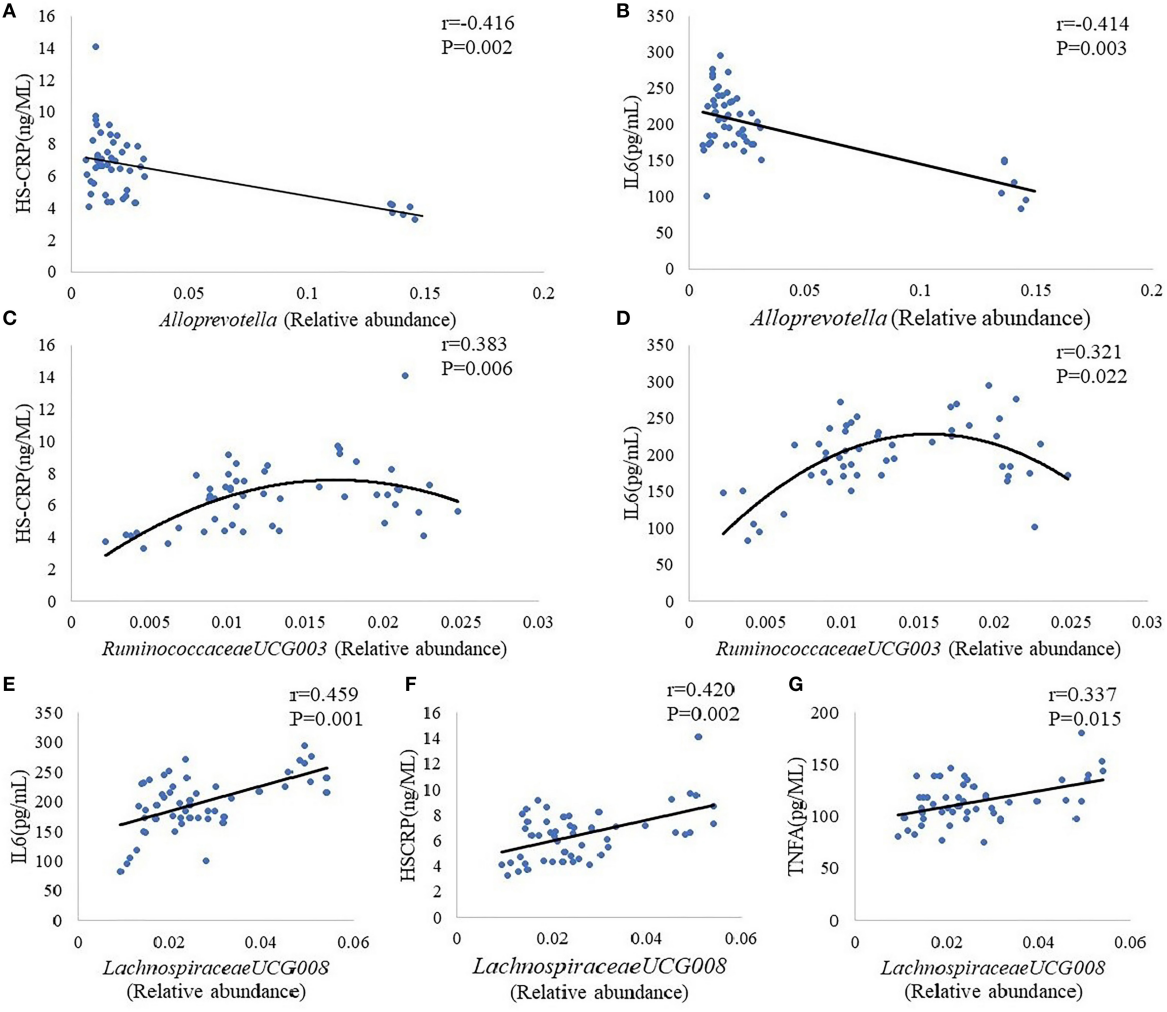
Correlation analysis between *Alloprevotella* and inflammatory markers (HS-CRP, IL-6) **(A,B)**. Correlation analysis between *Ruminococcaceae UCG-003* and inflammatory markers (HS-CRP, IL-6) **(C,D)**. Correlation analysis between *Lachnospiraceae UCG-008* and inflammatory markers (IL-6, HS-CRP, TNF-α) **(E–G)**. The “*r*” refers to the Spearman correlation coefficient, and the *P* < 0.05 indicate differences are significant.

*Alloprevotella, Lachnospiraceae UCG-008, Lachnospiraceae NK4A136, Lactobacillus, Ruminiclostridium 9*, and *Ruminococcaceae UCG-003* were choose for further analysis on the basic of results that the abundance of these five intestinal bacteria showed a significant difference between group M and group K. Results indicate the plasma concentration of IL-6,HS-CRP, TNF-α was associated with the abundance of *Alloprevotella, Lachnospiraceae UCG-008, Ruminococcaceae UCG-003*: there was a negative correlation between *Alloprevotella* and inflammatory markers (IL-6,HS-CRP) (Figures 5A,B); a positive correlation can be found in *Ruminococcaceae UCG-003* and inflammatory markers (IL-6,HS-CRP) (Figures 5C,D), as well as in *Lachnospiraceae UCG-008* and inflammatory markers (IL-6, HS-CRP, TNF-α) (Figures 5E–G).

#### Guizhi Fuling Wan Treatment Alter the Relative Abundance of Multiple Intestinal Flora

Combining the results given above, we chose *Alloprevotella, Ruminococcaceae UCG-003*, and *Lachnospiraceae UCG-008* to deeply discuss Guizhi Fuling Wan alleviate inflammation and improve insulin resistance by changing the structure of intestinal flora: in G group, the relative abundance of *Alloprevotella* was increased significantly, and *Ruminococcaceae UCG-003, Lachnospiraceae UCG-008* was decreased significantly (*P* < 0.05), compared with M group (Table 5). On the other hand, the effect of metformin (insulin sensitizer) on the relative abundance of the three bacteria was not significant (Table 5).

**Table 5:** The relative abundance of selected bacterial in low, medium and high dose of Guizhi Fuling Wan group, model group, and positive drug group.

	**Classification**	**M**	**D**	**Z**	**G**	**Y**
Lachnospiraceae UCG-008	Genus	0.0489 ± 0.0042	0.0232 ± 0.0048^Δ^	0.0154 ± 0.0016^*^	0.0229 ± 0.0018^Δ^	0.0294 ± 0.0018
Ruminococcaceae UCG-003	Genus	0.0187 ± 0.0021	0.0103 ± 0.0011	0.0116 ± 0.0015	0.0090 ± 0.0012^*^	0.0214 ± 0.0015
Alloprevotella	Genus	0.0112 ± 0.0011	0.0146 ± 0.0015	0.0189 ± 0.0023	0.0266 ± 0.0031^*^	0.0079 ± 0.0016

Compared with model group

* *P < 0.01*,

Δ *P < 0.05*.

*Blank control group (K), model control group (M), low dose of Guizhi Fuling Wan group (D), middle dose of Guizhi Fuling Wan group (Z), high dose of Guizhi Fuling Wan group (G), and positive drug (Metformin) control group (Y)*.

## Discussion

Polycystic ovary syndrome (PCOS) is a complex multi-organ disorder associated with metabolic disorder as well as inflammation, seriously affecting the physical, and mental health of affected women. The gut is the largest microecological environment in the human body and the gut microbiota is regarded as an endocrine organ including the maintenance of energy homeostasis and host immunity, the imbalance of gut microbiota has a decisive influence on human health including metabolism and immunity of the body (31).

The increasing knowledge of the role of microbiota in PCOS has provided new perspectives and methods to understand and treat PCOS (32, 33). Yanjie et al. found excess androgen production in PCOS rat model is related with dysbiosis of gut microbiota (16). Besides being related to the changes of serum hormone of PCOS, gut microbiota is also associated with occurrence of insulin resistance. As mentioned earlier, Bäckhed et al. had proved the correlation between gut microbiota and insulin resistance via fecal microbiota transplantation (FMT) (17). This study was later extended to humans, Kootte found at 6 weeks after lean donor (allogenic) fecal microbiota transplantation, insulin sensitivity of recipients with the metabolic syndrome was significantly improved, accompanied by altered microbiota composition. While no change can be observed in metabolism of recipients at 18 weeks after own (autologous) FMT (34). Collectively, these studies demonstrated the regulation of gut microbiota on insulin sensitivity. Reduced insulin sensitivity can lead to insulin resistance and compensatory hyperinsulinemia. Emerging as an important contributor to PCOS, on the one hand, IR can promote the luteinizing hormone (LH) and androgen secretion, reduce the content of serum sex hormone binding protein and aggravate hyperandrogenemia and endocrine disorder, on the other hand, it also can directly induce abnormal follicular development and atresia, causing polycystic ovarian morphology (35, 36). Inflammation is an established factor in the etiopathogenesis of insulin resistance. Groot had mentioned that studies in the early twentieth century with the results that large doses of salicylate can reduce glycosuria in patients, provided early clues that insulin resistance secondary to inflammation (37). Escobar summarize from the literatures that the chronic low-grade inflammation of PCOS woman is a key contributor to IR in PCOS (8). What's more, the mechanism of gut microbiota mediating insulin resistance lies in gut microbial-derived inflammatory responses (38).

Regarding PCOS, there is no record in ancient books of traditional Chinese medicine (TCM), but since the 1980s, the research on traditional Chinese medicine and PCOS has emerged (39). Guizhi Fuling Wan, a traditional Chinese formula with good effects of anti-inflammatory as well as improving IR, is widely used to treat PCOS in China, but whether these effects of Guizhi Fuling Wan are related to regulating gut flora is unclear. In this study, we chose letrozole, a non-steroidal aromatase inhibitor, combined with high-fat emulsion to induce PCOS rat model for investigating the association of Guizhi Fuling Wan alleviating inflammation and improving IR with gut microbiome. Firstly, the results of elevated plasma testosterone and INS, disturbed estrous cycle and polycystic change of ovarian tissue in M group were consistent with the typical characteristics of PCOS-IR, indicating that modeling successfully.

In the letrozole-induced PCOS model rats, Kelley et al. (40) found that the relative abundance of *Firmicutes, Lachnospiraceae*, and *Ruminococcaceae* is increased, while the relative abundance of *Bacteroidetes* is decreased. Our results are basically consistent with those results mentioned above: compared with K group, the relative abundance of *Alloprevotella* was decreased significantly, while the relative abundance of *Lachnospiraceae UCG-008, Lachnospiraceae NK4A136, Lactobacillus, Ruminiclostridium 9*, and *Ruminococcaceae UCG-003* was increased significantly. Besides altered gut microbiome, the concentrations of plasma INS, IL-6, HS-CRP, and TNF-α are increased in M group. In order to further analyze the gut microbiome mediated insulin resistance by inducing inflammation, we also analyzed the correlation between inflammatory factors and gut microbiome, as well as the correlation between inflammatory factors and fasting insulin. *Alloprevotella* is short-chain fatty acids (SCFAs)-producing bacteria (41). The SCFAs are the end products of dietary fiber fermented by the gut microbiota, having strong anti-inflammatory effects (42). Therefore, the *Alloprevotella* has the same effect on anti-inflammation with SCFAs, and our results confirm this conclusion. Our results presented that there was a negative correlation between *Alloprevotella* and inflammatory markers (IL-6, HS-CRP), which indicates that the concentrations of plasma IL-6, HS-CRP will decrease with the increase of the number of *Alloprevotella*. On the contrary, a positive correlation can be found in *Ruminococcaceae UCG-003* and inflammatory markers (IL-6, HS-CRP), as well as in *Lachnospiraceae UCG-008* and inflammatory markers (IL-6, HS-CRP, TNF-α), suggesting that reducing the number of *Ruminococcaceae UCG-003* and *Lachnospiraceae UCG-008* has certain benefits for the control of inflammation. However, previous literatures reported that *Lachnospiraceae* and *Ruminococcaceae* attenuates inflammation, the anti-inflammatory effect of *Ruminococcaceae* is dependent on secondary bile acids which can be produced by *Ruminococcaceae* (43, 44), the reason probably lies in the effect of *Lachnospiraceae* and *Ruminococcaceae* on anti-inflammatory may be species and strain specific. In addition, the serum fasting insulin concentration increased with the increase of il-6 and HS-CRP, indicating that *Ruminococcaceae UCG-003* and *Lachnospiraceae UCG-008* may have a positive relationship with serum fasting insulin concentration, and the *Alloprevotella* may have a negative relationship with serum fasting insulin concentration. Based on our results, Guizhi Fuliing Wan can increase the relative abundance of *Alloprevotella*, decrease the relative abundance of *Ruminococcaceae UCG-003* and *Lachnospiraceae UCG-008*. Simultaneously, Guizhi Fuliing Wan can also inhibit the release of inflammatory factors and improve insulin sensitivity. Considering of the correlation between the three kinds of bacteria and inflammatory factors, and the correlation between inflammatory factors and fasting INS, thus we have a reason to believe Guizhi Fuliing Wan is capable to regulate gut microbiome positively to reduce the release of inflammatory factors and achieves the goal of improving insulin resistance ultimately.

In conclusion, we found Guizhi Fuling Wan can regulate the structure of gut microbiome, and this regulatory effect of Guizhi Fuling Wan is the basis of alleviating inflammation and improving insulin resistance. Our study provides a basis for promoting the treatment of PCOS with Guizhi Fuling Wan and lays a solid foundation for the treatment of PCOS with TCM. Recently, Qi et al. (45) found that bile acid, a metabolic product of intestinal bacteria, is involved in improving PCOS-IR. Our present results showed that Guizhi Fuling Wan could reshape the intestinal flora. Whether Guizhi Fuling Wan can interfere with the generation of bile acids through regulating intestinal flora remain unclear. If possible, whether interfering with bile acid metabolism is another way of Guizhi Fuling Wan to improve insulin resistance, which needs further experiments to verify.

## Data Availability Statement

The datasets presented in this study can be found in online repositories. The names of the repository/repositories and accession number(s) can be found in the article/Supplementary Material.

## Ethics Statement

This animal study was reviewed and approved by the Animal Care Committee of Chengdu University of TCM, China (SYXK2018-0126).

## Author Contributions

HZ, YL, ML, YZ, and XH contributed conception and design of the study. YL and ML organized the database. YZ performed the statistical analysis and wrote the first draft of the manuscript. All authors contributed to manuscript revision, read, and approved the submitted version.

## Conflict of Interest

The authors declare that the research was conducted in the absence of any commercial or financial relationships that could be construed as a potential conflict of interest.

## References

[1] Torres PJ Ho BS Arroyo P Sau L Chen A Kelley ST. Exposure to a healthy gut microbiome protects against reproductive and metabolic dysregulation in a PCOS mouse model. Endocrinology. (2019) 160:1193–204. 10.1210/en.2019-00050 30924862 PMC6482036

[2] Escobar-Morreale HF. Polycystic ovary syndrome: definition, aetiology, diagnosis and treatment. Nat Rev Endocrinol. (2018) 14:270–84. 10.1038/nrendo.2018.24 29569621

[3] Li R Zhang Q Yang D Li S Lu S Wu X. Prevalence of polycystic ovary syndrome in women in China: a large community-based study. Hum Reprod. (2013) 28:2562–9. 10.1093/humrep/det262 23814096

[4] Azziz R. Polycystic ovary syndrome. Obstet Gynecol. (2018) 132:321–36. 10.1097/aog.0000000000002698 29995717

[5] Prapas N Karkanaki A Prapas I Kalogiannidis I Katsikis I Panidis D. Genetics of polycystic ovary syndrome. Expert Rev Mol Diagnost. (2017) 17:723–33. 10.1080/14737159.2017.1340833 20011085 PMC2776334

[6] Fields EL Trent ME. Treatment considerations for the cardiometabolic signs of polycystic ovary syndrome: a review of the literature since the 2013 endocrine society clinical practice guidelines. JAMA Pediatrics. (2016) 170:502–7. 10.1001/jamapediatrics.2015.4866 27018935

[7] Stepto NK Moreno-Asso A McIlvenna LC Walters KA Rodgers RJ. Molecular mechanisms of insulin resistance in polycystic ovary syndrome: unraveling the conundrum in skeletal muscle? J Clin Endocrinol Metab. (2019) 104:5372–81. 10.1210/jc.2019-00167 30938770

[8] Escobar-Morreale HF Luque-Ramírez M González F. Circulating inflammatory markers in polycystic ovary syndrome: a systematic review and meta analysis. Fertility Sterility. (2011) 95:1048–58.e1-2. 10.1016/j.fertnstert.2010.11.036 21168133 PMC3079565

[9] Norman RJ Dewailly D Legro RS Hickey TE. Polycystic ovary syndrome. Lancet. (2007) 370:685–97. 10.1016/S0140-6736(07)61345-2 17720020

[10] González F Sia CL Shepard MK Rote NS Minium J. Inflammation in response to glucose ingestion is independent of excess abdominal adiposity in normal-weight women with polycystic ovary syndrome. J Clin Endocrinol Metab. (2012) 97:4071–9. 10.1210/jc.2012-2131 22904174 PMC3485595

[11] Lindheim L Bashir M Münzker J Trummer C Zachhuber V Leber B. Alterations in gut microbiome composition and barrier function are associated with reproductive and metabolic defects in women with Polycystic Ovary Syndrome (PCOS): a pilot study. PLoS ONE. (2017) 12:e0168390. 10.1371/journal.pone.0168390 28045919 PMC5207627

[12] Diamanti-Kandarakis E Dunaif A. Insulin resistance and the polycystic ovary syndrome revisited: an update on mechanisms and implications. Endocr. Rev. (2012) 33:981–1030. 10.1210/er.2011-1034 23065822 PMC5393155

[13] Çakiroglu Y Vural F Vural B. The inflammatory markers in polycystic ovary syndrome: association with obesity and IVF outcomes. J Endocrinol Invest. (2016) 39:899–907. 10.1007/s40618-016-0446-4 26980590

[14] Kelly CC Lyall H Petrie JR Gould GW Connell JM Satt N. Low grade chronic inflammation in women with polycystic ovarian syndrome. J Clin Endocrinol Metab. (2001) 86:2453–5. 10.1210/jcem.86.6.7580 11397838

[15] Liu R Zhang C Shi Y Zhang F Li L Wang X. Dysbiosis of gut microbiota associated with clinical parameters in polycystic ovary syndrome. Front. Microbiol. (2017) 8:324. 10.3389/fmicb.2017.00324 28293234 PMC5328957

[16] Yanjie G Yane Q Xuefei Y Lihui Z Shu W Yinhui L. Association between polycystic ovary syndrome and gut microbiota. PLoS ONE. (2016) 11:e0153196. 10.1371/journal.pone.0153196 27093642 PMC4836746

[17] Bäckhed F Ding H Wang T Hooper LV Koh GY Nagy A. The gut microbiota as an environmental factor that regulates fat storage. Proc Natl Acad Sci USA. (2004) 101:15718–23. 10.1073/pnas.0407076101 15505215 PMC524219

[18] Wen L Duffy A. Factors influencing the gut microbiota, inflammation, and type 2 diabetes. J Nutr. (2017) 147:1468–75S. 10.3945/jn.116.240754 28615382 PMC5483960

[19] Meng W Ta N Wang F. Add-on effect of Guizhi Fuling formula to mifepristone for endometriosis: a meta-analysis of randomized controlled trials. Medicine. (2019) 98:e16878. 10.1097/md.0000000000016878 31415429 PMC6831320

[20] Lixin Z Clinical study on treatment of polycystic ovary syndrome with insulin resistance with berberine and cassia twig tuckahoe pill. Shaanxi Zhongyi. (2018) 39:602–4. 10.3969/j.issn.1000-7369.2018.05.018

[21] Qiusheng Z Xiufen T Nansu W Effect of cassia fuling pill on insulin resistance and adiponectin in rats with polycystic ovary syndrome. J New Chin Med. (2012) 44:116–7.

[22] Zhang LS Yang FW Zhang JH Zheng WK Zhang MY Li Y. [Guizhi Fuling capsule/pill treatment for chronic pelvic inflammatory disease: a systematic review of randomized clinical trials]. Zhongguo Zhong Yao Za Zhi. (2017) 42:1500–9. 10.19540/j.cnki.cjcmm.2017.0048 29071853

[23] Yue W Clinical observation on the treatment of polycystic ovary syndrome with Cassia twig tuckahoe pill. Shandong Med J. (2006) 46:70–1. 10.3969/j.issn.1002-266X.2006.01.054

[24] Ong M Peng J Jin X Qu X. Chinese herbal medicine for the optimal management of polycystic ovary syndrome. Am J Chin Med. (2017) 45:405–22. 10.1142/s0192415x17500252 28359195

[25] Li YC Qiao JY Wang BY Bai M Shen JD Cheng YX. Paeoniflorin ameliorates fructose-induced insulin resistance and hepatic steatosis by activating LKB1/AMPK and AKT pathways. Nutrients. (2018) 10:1024. 10.3390/nu10081024 30081580 PMC6116094

[26] Ma Z Liu H Wang W Guan S Yi J Chu L. Paeoniflorin suppresses lipid accumulation and alleviates insulin resistance by regulating the Rho kinase/IRS-1 pathway in palmitate-induced HepG2Cells. Biomed Pharmacother. (2017) 90:361–7. 10.1016/j.biopha.2017.03.087 28380411

[27] Xiaojuan X Lijuan Y Zhang W Wanjing L Qiuxiang W Yunliang H Research on change of ovarian morphology in improved PCOS animal models which were stimulated to have feature information of kidney deficiency phlegm-damp stagnation. J Chengdu Univ TCM. (2016) 39:5–9, 14 10.13593/j.cnki.51-1501/r.2016.02.005

[28] Kafali H Iriadam M Ozardali I Demir N. Letrozole-induced polycystic ovaries in the rat: a new model for cystic ovarian disease. Archiv Med Res. (2004) 35:103–8. 10.1016/j.arcmed.2003.10.005 15010188

[29] Liu C Yao M Stegen JC Rui J Li J Li X. Long-term nitrogen addition affects the phylogenetic turnover of soil microbial community responding to moisture pulse. Sci Rep. (2017) 7:17492. 10.1038/s41598-017-17736-w 29235487 PMC5727477

[30] Caporaso JG Lauber CL Walters WA Berg-Lyons D Lozupone CA Turnbaugh PJ. Global patterns of 16S rRNA diversity at a depth of millions of sequences per sample. Proc Natl Acad Sci USA. (2011) 108(Suppl. 1) 4516–22. 10.1073/pnas.1000080107 20534432 PMC3063599

[31] Quaranta G Sanguinetti M Masucci L. Fecal microbiota transplantation: a potential tool for treatment of human female reproductive tract diseases. Front Immunol. (2019) 10:2653. 10.3389/fimmu.2019.02653 31827467 PMC6890827

[32] Insenser M Murri M Del Campo R Martínez-García MÁ Fernández-Durán E Escobar-Morreale HF. Gut microbiota and the polycystic ovary syndrome: influence of sex, sex hormones, and obesity. J Clin Endocrinol Metab. (2018) 103:2552–62. 10.1210/jc.2017-02799 29897462

[33] Moreno-Indias I Sánchez-Alcoholado L Sánchez-Garrido MÁ Martín-Núñez GM Pérez-Jiménez F Tena-Sempere M. Neonatal androgen exposure causes persistent gut microbiota dysbiosis related to metabolic disease in adult female rats. Endocrinology. (2016) 157:4888–98. 10.1210/en.2016-1317 27700135

[34] Kootte RS Levin E Salojärvi J Smits LP Hartstra AV Udayappan SD. Improvement of insulin sensitivity after lean donor feces in metabolic syndrome is driven by baseline intestinal microbiota composition. Cell Metab. (2017) 26:611–9.e6. 10.1016/j.cmet.2017.09.008 28978426

[35] Dumesic DA Oberfield SE Stener-Victorin E Marshall JC Laven JS Legro RS. Scientific statement on the diagnostic criteria, epidemiology, pathophysiology, and molecular genetics of polycystic ovary syndrome. Endocr Rev. (2015) 36:487–525. 10.1210/er.2015-1018 26426951 PMC4591526

[36] Yiyu W Ye L Hefeng H Roles of gut microbiota in the occurrence and development of polycystic ovary syndrome. J Shanghai Jiaotong Univ. (2018) 38:454–7. 10.3969/j.issn.1674-8115.2018.04.019

[37] de Groot PF Frissen MN de Clercq NC Nieuwdorp M. Fecal microbiota transplantation in metabolic syndrome: history, present and future. Gut Microbes. (2017) 8:253–67. 10.1080/19490976.2017.1293224 28609252 PMC5479392

[38] Khan MT Nieuwdorp M Bäckhed F. Microbial modulation of insulin sensitivity. Cell Metab. (2014) 20:753–60. 10.1016/j.cmet.2014.07.006 25176147

[39] Zhou K Zhang J Xu L Wu T Lim CE. Chinese herbal medicine for subfertile women with polycystic ovarian syndrome. Cochr Database Syst Rev. (2016) 10:CD007535. 10.1002/14651858.CD007535 27731904 PMC6457959

[40] Kelley ST Skarra DV Rivera AJ Thackray VG. The gut microbiome is altered in a letrozole-induced mouse model of polycystic ovary syndrome. PLoS ONE. (2016) 11:e0146509. 10.1371/journal.pone.0146509 26731268 PMC4701222

[41] Liu J Yue S Yang Z Feng W Meng X Wang A. Oral hydroxysafflor yellow A reduces obesity in mice by modulating the gut microbiota and serum metabolism. Pharmacol Res. (2018) 134:40–50. 10.1016/j.phrs.2018.05.012 29787870

[42] Richards JL Yap YA McLeod KH Mackay CR Mariño E. Dietary metabolites and the gut microbiota: an alternative approach to control inflammatory and autoimmune diseases. Clin Transl Immunol. (2016) 5:e82. 10.1038/cti.2016.29 27350881 PMC4910123

[43] Truax AD Chen L Tam JW Cheng N Guo H Koblansky AA. The inhibitory innate immune sensor NLRP12 maintains a threshold against obesity by regulating gut microbiota homeostasis. Cell Host Microbe. (2018) 24:364–78.e6. 10.1016/j.chom.2018.08.009 30212649 PMC6161752

[44] Sinha SR Haileselassie Y Nguyen LP Tropini C Wang M Becker LS. Dysbiosis-induced secondary bile acid deficiency promotes intestinal inflammation. Cell Host Microbe. (2020) 27:659–70.e5. 10.1016/j.chom.2020.01.021 32101703 PMC8172352

[45] Qi X Yun C Sun L Xia J Wu Q Wang Y Gut microbiota-bile acid-interleukin-22 axis orchestrates polycystic ovary syndrome. Nat Med. (2019) 25:1225–33. 10.1038/s41591-019-0509-0 31332392 PMC7376369

