# Supplementary figures and images for: Guizhi Fuling Wan, Chinese Herbal Medicine, Ameliorates Insulin Sensitivity in PCOS Model Rats With Insulin Resistance via Remodeling Intestinal Homeostasis
Source: Front Endocrinol (Lausanne). 2020 Aug 27;11:575. doi: 10.3389/fendo.2020.00575 (PMC7482315; doi:10.3389/fendo.2020.00575)

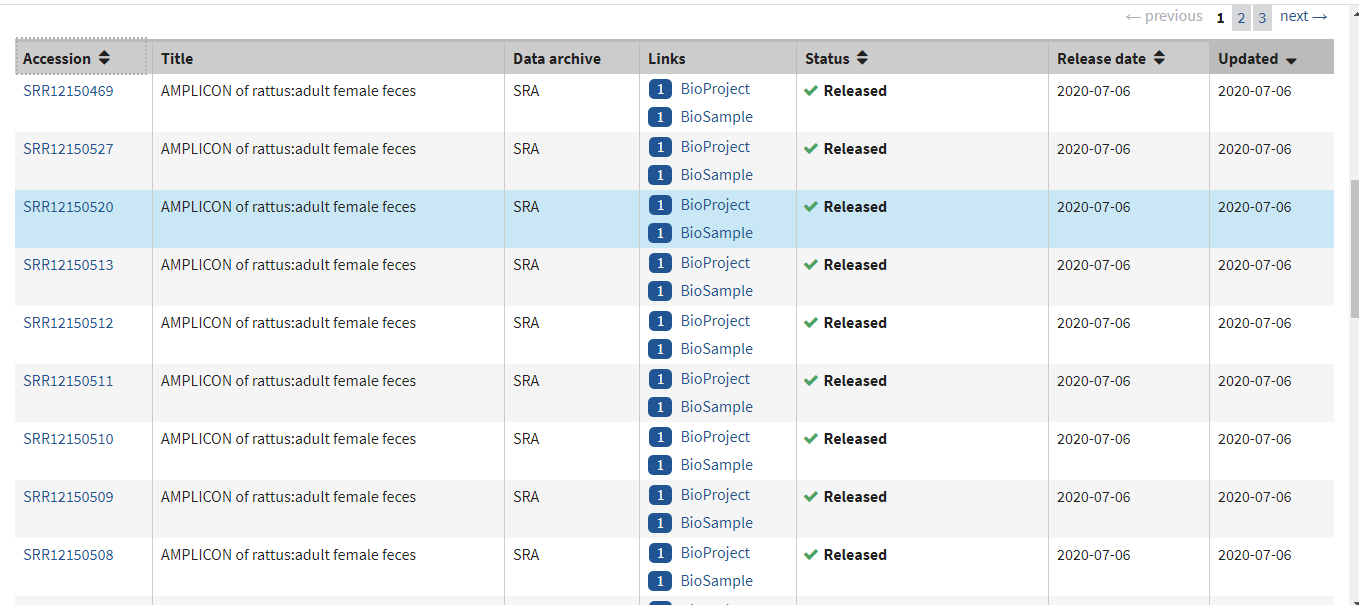

Supplement: Supplementary file 1 [file Image_1.PNG]

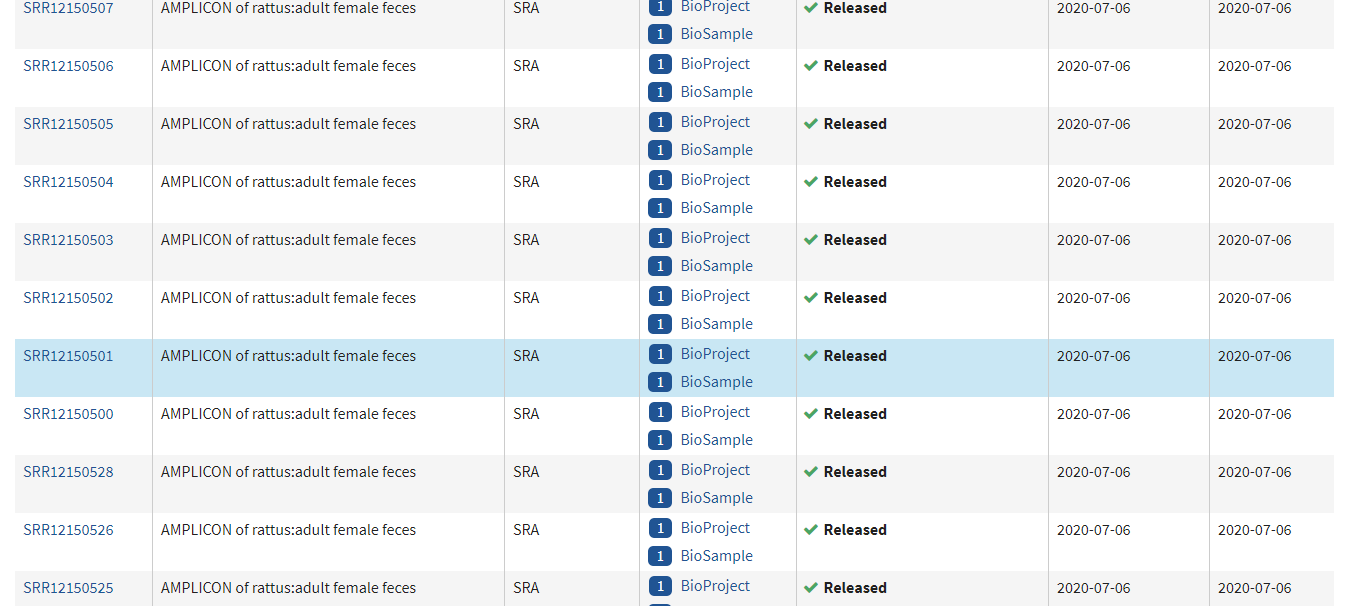

Supplement: Supplementary file 2 [file Image_2.PNG]

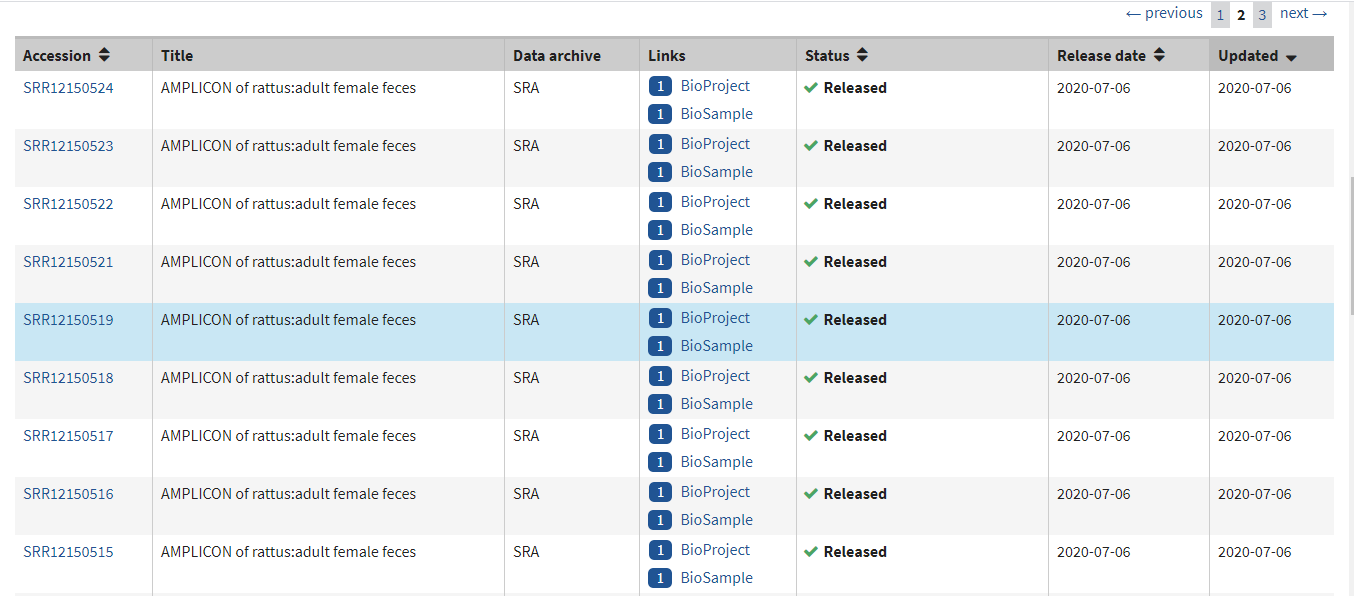

Supplement: Supplementary file 3 [file Image_3.PNG]

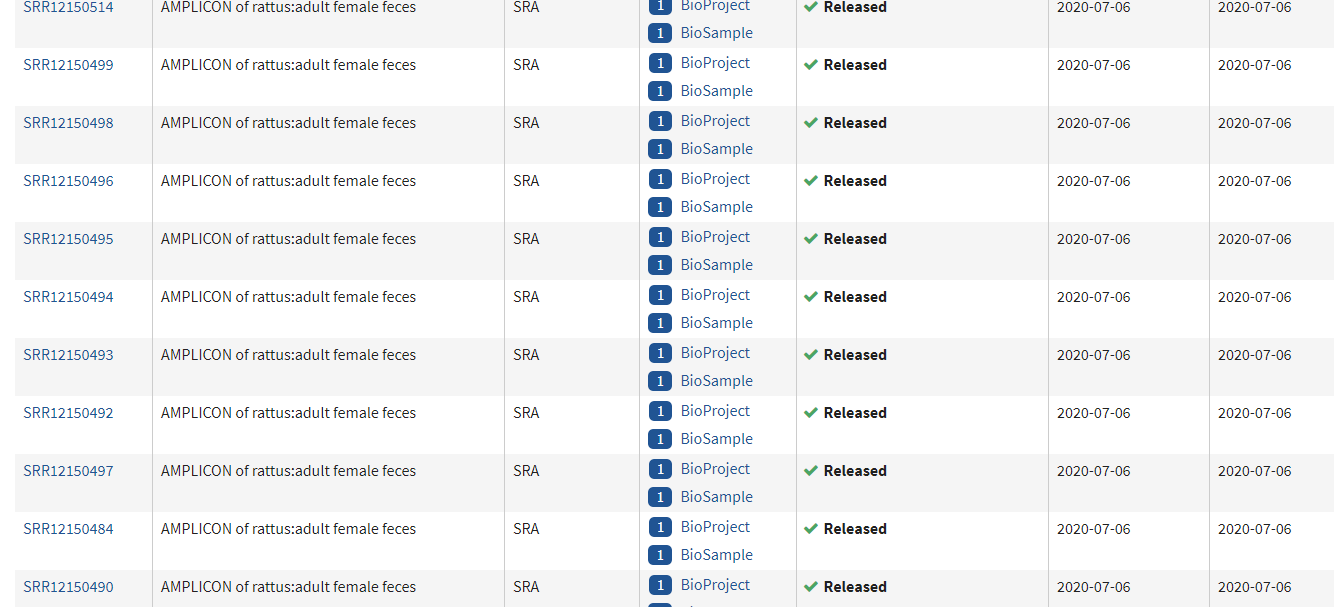

Supplement: Supplementary file 4 [file Image_4.PNG]

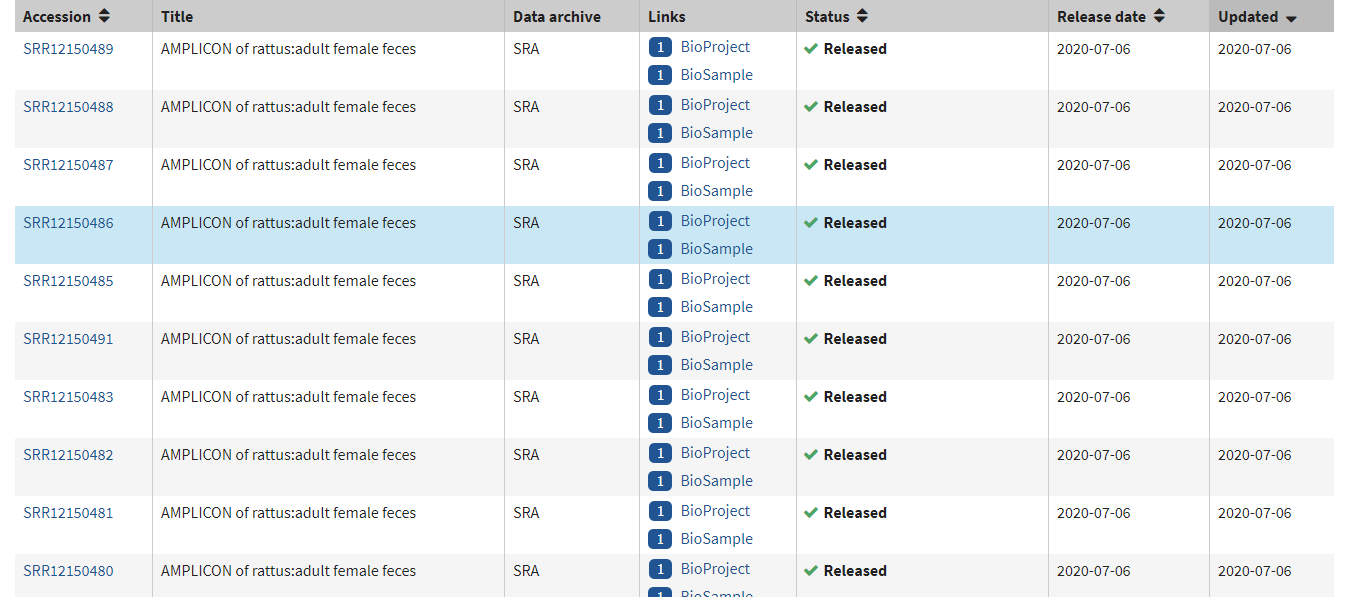

Supplement: Supplementary file 5 [file Image_5.PNG]

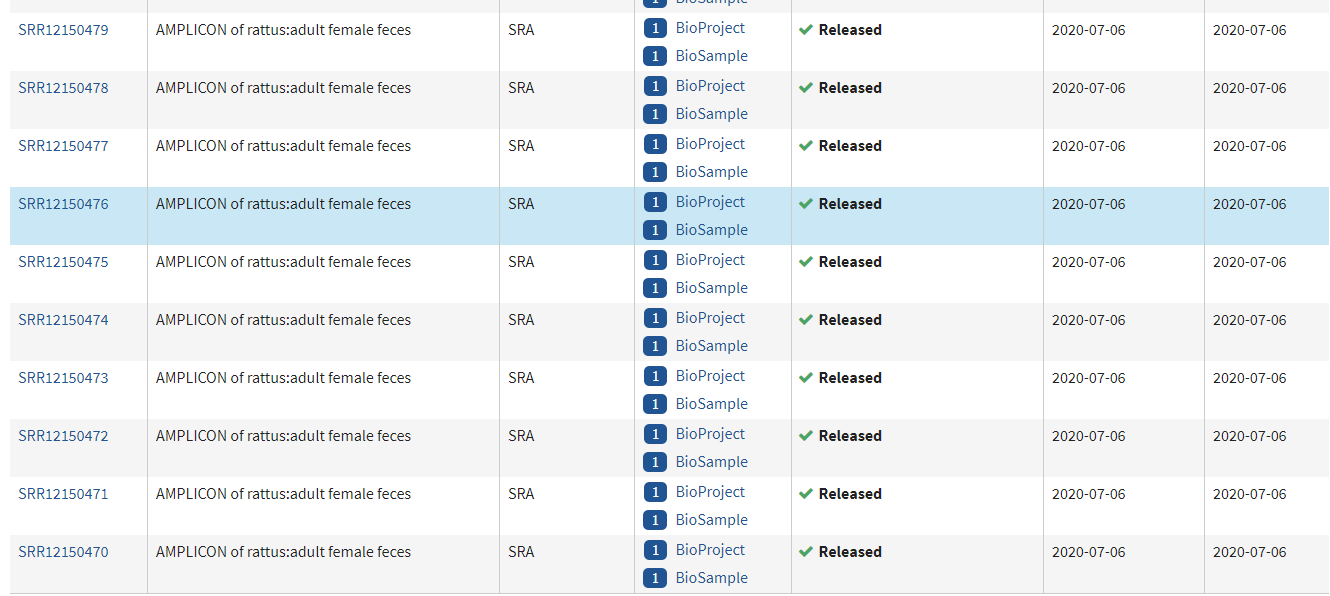

Supplement: Supplementary file 6 [file Image_6.PNG]
